# Malignant mesothelioma

**DOI:** 10.1186/1750-1172-3-34

**Published:** 2008-12-19

**Authors:** Alastair J Moore, Robert J Parker, John Wiggins

**Affiliations:** 1 Department of Respiratory Medicine, Wexham Park Hospital, Wexham, Slough, Berkshire, SL2 4HL, UK

## Abstract

Malignant mesothelioma is a fatal asbestos-associated malignancy originating from the lining cells (mesothelium) of the pleural and peritoneal cavities, as well as the pericardium and the tunica vaginalis. The exact prevalence is unknown but it is estimated that mesotheliomas represent less than 1% of all cancers. Its incidence is increasing, with an expected peak in the next 10–20 years. Pleural malignant mesothelioma is the most common form of mesothelioma. Typical presenting features are those of chest pain and dyspnoea. Breathlessness due to a pleural effusion without chest pain is reported in about 30% of patients. A chest wall mass, weight loss, sweating, abdominal pain and ascites (due to peritoneal involvement) are less common presentations. Mesothelioma is directly attributable to occupational asbestos exposure with a history of exposure in over 90% of cases. There is also evidence that mesothelioma may result from both para-occupational exposure and non-occupational "environmental" exposure. Idiopathic or spontaneous mesothelioma can also occur in the absence of any exposure to asbestos, with a spontaneous rate in humans of around one per million. A combination of accurate exposure history, along with examination radiology and pathology are essential to make the diagnosis. Distinguishing malignant from benign pleural disease can be challenging. The most helpful CT findings suggesting malignant pleural disease are 1) a circumferential pleural rind, 2) nodular pleural thickening, 3) pleural thickening of > 1 cm and 4) mediastinal pleural involvement. Involvement of a multidisciplinary team is recommended to ensure prompt and appropriate management, using a framework of radiotherapy, chemotherapy, surgery and symptom palliation with end of life care. Compensation issues must also be considered. Life expectancy in malignant mesothelioma is poor, with a median survival of about one year following diagnosis.

## Disease name and definition

Malignant mesothelioma is a cancer originating from the lining cells (mesothelium) of the pleural and peritoneal cavities, as well as the pericardium and the tunica vaginalis [1]. Its distribution may be uni- or multifocal or may involve the lining cells in a continuous manner.

## Epidemiology and aetiology

Before 1950, malignant mesothelioma was so rare that some pathologists even questioned its existence [2]. However, the increasing use of asbestos after the second world war led to the description of a causal relationship between asbestos exposure and the development of mesothelioma in 1960 [3]. Although its use was widely abandoned in the western world in the 1980s, the long latency period between exposure to asbestos and onset of mesothelioma, which can range from 15 to 60 years [4,5], meant that the mortality rates from mesothelioma have continued to rise. In the USA the annual deaths from mesothelioma peaked at 3060 in 2002 and have subsequently declined, however the incidence of mesothelioma will continue to rise in the UK until it reaches a peak in about the year 2020 [6]. According to data gathered in the US Surveillance Epidemiology and End Results programme for 1973–1992, there has been virtually a constant rate of mesothelioma in females but a consistently higher rate for males. In the 1960s a mesothelioma register was set up in the UK to systematically record the mortality rates from mesothelioma and to try to identify the incidence of tumour development without known occupational exposure. It is predicted that around 90,000 deaths will occur from mesothelioma by 2050, with 65,000 of these occurring from 2002 onwards [7].

Asbestos is a naturally occurring fibrous silicate, and the risk of developing mesothelioma depends on the exposure to different types of the asbestos mineral fibre. The main asbestos mineral groups are serpentine fibres, which are long and curly, or amphibole fibres which are straight and rod-like. This distinction is important as the serpentine fibre shape is more easily cleared from the respiratory tract. Epidemiologic data suggests that the amphibole, crocidolite, is associated with the highest risk of mesothelioma [8] and that the serpentine fibre, chrysotile has the lowest. The diagnosis of mesothelioma is directly attributable to occupational asbestos exposure; however there is evidence that mesothelioma may result from both para-occupational exposure (*e.g*.: women having laundered their husband's overalls) and non-occupational "environmental" exposure [9]. Idiopathic or spontaneous mesothelioma can also occur in the absence of any exposure to asbestos in both animals [10] and humans [8], and a recent review suggests a spontaneous mesothelioma rate in humans of around one per million [11].

Non-asbestos mineral fibres have also been shown to induce mesothelioma, such as erionite found in certain areas of Turkey [12] or tremolite in north-western Greece [13], and whilst not specifically mined for commercial purposes, tremolite is often found as a contaminant of chrysotile asbestos and has been causally linked with an increased risk of mesothelioma [14]. The role of Simian virus (SV-40) in the pathogenesis of mesothelioma is more controversial; SV-40 was found to contaminate polio vaccines in the 1950s and 60s in the UK, and although it has been suggested that it is a causative factor in the development of mesothelioma [15-17], recent studies have found no link [18-20].

## Clinical description

Mesothelioma is primarily a disease of adults and usually presents in the fifth to seventh decades, and 70–80% of cases occur in men. Those diagnosed between the ages of 20 to 40 years usually have a history of childhood exposure [1]. Typical presenting features are those of chest pain, dyspnoea or both [11,21], and in one series up to a third of patients presented with breathlessness due to a pleural effusion without chest pain [22]. When it occurs, the chest pain tends to be dull or boring in nature, but a pleuritic-type pain can occur in the presence of pleural effusions. Involvement of the mediastinal structures is well recognized but hoarseness of the voice and superior vena caval obstruction only rarely causes major symptoms. Dysphagia can also occur but this is a late finding. Unlike bronchogenic carcinomas, presentation with haemoptysis, lymphadenopathy and metastatic symptoms are unusual. A chest wall mass, weight loss and sweating are less common presentations, as is peritoneal mesothelioma although involvement may be found in up to one third of cases at autopsy. Presentation of peritoneal mesothelioma is with non-specific symptoms including loss of appetite, nausea and vomiting, diarrhoea or constipation and occasionally ascites. Small bowel obstruction is usually a late feature, and overall the prognosis of peritoneal mesothelioma is worse than pleural mesothelioma with a mean survival time of about 7 months [11].

Physical examination is usually unremarkable except for signs of pleural effusion and pleural thickening due to tumour infiltration. Finger clubbing is more common than in benign asbestos related pleural disease, and can occur in up to 30% of cases [23]. The tumour originates mainly on the parietal pleura and spreads via the fissures, to encase the lung surfaces. Infiltration of the pericardium can result in signs of cardiac tamponade, and mesothelioma can grow along needle tracks and incisions. Blood tests can reveal an elevated erythrocyte sedimentation rate (ESR) [24], and there have been isolated case reports of mesothelioma associated with autoimmune haemolytic anaemia [25].

## Diagnosis and differential diagnosis

The combination of accurate history, examination, radiology and the acquisition of pathology is essential in the diagnosis of mesothelioma. A careful history of asbestos exposure is essential, and the identification of at-risk occupations are strong markers of exposure. However, the delay between exposure and presentation may naturally preclude accurate recall of occupational exposure and working conditions which may have occurred up to 60 years previously.

In those patients with a pleural effusion, sampling of the fluid for cytological examination is the first step in confirming the diagnosis. Pleural fluid cytology is positive for malignant cells in about a third of cases [1] and if the clinical, radiological and cytological results support a diagnosis of mesothelioma then this can be accepted. However, it is uncommon for the definitive diagnosis to be made on pleural fluid cytology alone and pleural biopsy for tissue diagnosis is therefore recommended. A contrast enhanced computed tomogram (CT) scan is essential to both identify the extent of the disease, and help guide a percutaneous biopsy if the pleural fluid cytological analysis is not sufficient.

### Radiological findings

Radiological imaging is essential for the diagnosis, staging and management of mesothelioma. X-ray, CT, magnetic resonance imaging (MRI) and positron emission tomography (PET) have all been used to evaluate the disease.

#### CT

Intravenous contrast-enhanced CT is the primary imaging modality for suspected pleural malignant disease. CT allows visualisation of the whole pleural surface and diaphragm and use of a 45–60 second scan delay enables the pleural surfaces to be studied whilst still allowing assessment of the mediastinal nodes [26]. A standard protocol should include the liver and adrenal glands, but in cases where there is a past history of abdominal or pelvic malignancy, the scan should also include the lower abdomen and pelvis [11].

Distinguishing malignant from benign pleural disease can be challenging. The most helpful CT findings suggesting malignant pleural disease are 1) a circumferential pleural rind, 2) nodular pleural thickening, 3) pleural thickening of > 1 cm and 4) mediastinal pleural involvement [27]. The specificities of these findings were 100%, 94%, 94% and 88% respectively. The sensitivities were 41%, 51%, 36% and 56% respectively. The presence of bilateral pleural calcification on CT is uncommon in malignant mesothelioma [27]. A significant reduction in thoracic volume seen on CT is more common, however, occurring in up to 73% of cases according to some series [28]. Whilst these features have a high positive predictive value, absence of these signs does not reliably exclude the diagnosis of pleural malignancy.

#### MRI

MRI screening is not used routinely in the assessment of malignant mesothelioma, however in patients with potentially resectable disease, MRI can help to provide additional staging information over and above CT. Using gadolinium enhancement, MRI can improve the identification of tumour extension into the diaphragm or chest wall, allowing better assessment of the individual for surgical treatment. MRI also is the imaging modality of choice in those in whom intravenous iodinated contrast is contraindicated [29].

#### PET

The standardized uptake value (SUV) in PET is a semi-quantitative measure of the metabolic activity of a lesion and the SUV is significantly higher in mesothelioma than in other benign pleural diseases such as pleural plaques or inflammatory pleuritis [29,30], and one study found PET scanning to have a 96.8% sensitivity and an 88.5% specificity at distinguishing benign from malignant pleural disease [31]. PET scanning has also increased the accuracy in diagnosing mediastinal nodal metastases [30] and therefore the combination of metabolic and anatomical information provided by PET makes it useful in the staging and preoperative evaluation of mesothelioma. PET may also help as a guide to the optimal site for CT guided pleural biopsy, and there is evidence that changes in the fluorodeoxyglucose (FDG) uptake within the tumour might indicate response to treatment suggesting its role in the assessment of response to both chemotherapy and chemo-radiotherapy [32].

#### Staging and assessment of disease response

At least six different staging systems have been suggested for malignant mesothelioma, but none have been accurately shown to predict survival. Currently, a TNM staging system (Table 1) similar to that used in non-small cell lung carcinoma has been proposed by the International Mesothelioma Interest Group (IMIG) [33].

**Table 1:** IMIG TNM staging system

**Primary tumour (T)**	
T1a	Tumour limited to the ipsilateral parietal including mediastinal and diaphragmatic pleura, no involvement of the visceral pleura

T1b	Tumour involving the ipsilateral parietal including mediastinal and diaphragmatic pleura, scattered foci of tumour also involving the visceral pleura

T2	Tumour involving each of the ipsilateral pleural surfaces (parietal, mediastinal, diaphragmatic and visceral pleura) with at least one of the following features: • involvement of diaphragmatic muscle • confluent visceral pleural tumour (including the fissures) • extension of tumour from visceral pleura into the underlying pulmonary parenchyma

T3	Locally advanced but potentially resectable tumour; tumour involving all of the ipsilateral pleural surfaces (parietal, mediastinal, diaphragmatic and visceral pleura) with at least one of the following features: • involvement of the endothoracic fascia • extension into the mediastinal fat • solitary, completely resectable focus of tumour extending into the soft tissues of the chest wall • non transmural involvement of the pericardium

T4	Locally advanced technically unresectable tumour; tumour involving all of the ipsilateral pleural surfaces (parietal, mediastinal, diaphragmatic and visceral) with at least one of the following features: • diffuse extension or multifocal masses of tumour in the chest wall with or without associated rib destruction • direct transdiaphragmatic extension of tumour to the peritoneum • direct extension of tumour to the contralateral pleura • direct extension of tumour to one or more mediastinal organs • direct extension of tumour into the spine: tumour extending through to the internal surface of the pericardium with or without a pericardial effusion • tumour involving the myocardium

**Lymph nodes (N)**	

Nx	Regional lymph nodes cannot be assessed

N0	No regional lymph node metastases

N1	Metastases in the ipsilateral bronchopulmonary or hilar lymph nodes

N2	Metastases in the subcarinal or the ipsilateral mediastinal lymph nodes including the ipsilateral internal mammary nodes

N3	Metastases in the contralateral mediastinal, contralateral internal mammary, ipsilateral or contralateral supraclavicular lymph nodes

**Metastases (M)**	

Mx	Presence of distant metastases cannot be assessed

M0	No distant metastasis

M1	Distant metastasis present

Tumour response to treatment is an important surrogate for patient benefit. The World Health Organization (WHO) criteria for tumour response were most useful for measuring bi-dimensional lesions, whereas the irregular growth pattern of mesothelioma as a rind around the chest wall makes these criteria poorly applicable [34]. More recently, the Response Evaluation Criteria in Solid Tumours (RECIST) [35] uses a uni-dimensional measurement of tumour size to assess response, but this is based on the assumption that tumours are largely spherical, so again there are limitations to the applicability of this technique in the assessment of malignant mesothelioma.

A modified RECIST criteria has now been published, however, with particular reference to malignant mesothelioma [36]. Assessment of response to treatment is now made by measuring uni-dimensional tumour thickness perpendicular to the chest wall in 2 sites at three different levels on CT.

### Pathological findings

According to the WHO classification, malignant mesothelioma can be classified as epithelioid, sarcomatoid, or biphasic based on tissue obtained by biopsy. The pathological diagnosis is reached with the aid of immunohistochemistry to demonstrate the presence of mesothelial, epithelial, or true sarcomatous differentiation in the malignant cells [37]. The reported diagnostic yield from CT guided biopsy varies from 60% with a single attempt up to 85% with multiple attempts [38]. Ultimately, however, the highest yields are obtained with open or thoracoscopic pleural biopsy.

There is currently no individual immunohistochemical mesothelial marker that provides 100% specificity and high sensitivity for the diagnosis of malignant mesothelioma [37] and so a number of mesothelial and epithelial markers have been developed. Clearly, distinguishing a malignant pleural process from an inflammatory process is a priority and immunohistochemistry has demonstrated that mesothelioma frequently shows immunoreactivity for keratin, p53 and epithelial membrane antigen (EMA) which is unusual for benign pleural disease.

Differentiating malignant mesothelioma from other malignancies, however, can be more difficult as mesotheliomas, especially those of the epithelioid type, can mimic several other tumours. Again, immunohistochemistry can help in the differentiation of mesothelioma and markers such as mesothelin, cytokeratin 5/6, calretinin, thrombomodulin and WT-1 have been used. The most specific and sensitive markers for mesothelioma are mesothelin (in epithelioid mesothelioma), calretinin and cytokeratin 5/6. However, according to a recent review article, mesothelin is positive in 27% of squamous cell carcinomas, and calretinin and cytokeratin 5/6 can be raised in both squamous cell carcinoma of the lung and adenocarcinoma of both the lung and other sites [37]. Clearly there can be difficulty in making the diagnosis, and so usually a panel of two positive and two negative markers will be sufficient to confirm the presence of malignant mesothelioma.

#### Serum markers in the diagnosis of mesothelioma

Reliably diagnosing malignant mesothelioma early in the disease is notoriously difficult due to the variability in time to presentation from exposure in the disease. Recently there has been much interest in the use of serum markers for the diagnosis of the disease. The ideal serum marker would be one that is able to offer: 1) early diagnosis, 2) identification of all the subtypes, 3) differentiation of malignant mesothelioma from benign pleural disease and other metastatic pleural malignancies, and 4) be able to track response to therapy and predict survival. As yet no such ideal marker exists, but studies have suggested the use of osteopontin, mesothelin or megakaryocyte potentiating factor in this role.

Osteopontin is a glycoprotein that is over-expressed in lung, breast, colorectal, gastric and ovarian carcinomas and in melanoma. Increased levels correlate with tumour progression, invasion and metastases. Although increased levels do not exclude other malignancies, recent data suggest that osteopontin has great potential use as a marker for mesothelioma [39] and has a positive predictive power for mesothelioma equivalent to Ca-125 for ovarian cancer. Studies have suggested a sensitivity and specificity for mesothelioma of 77% and 85% respectively [40].

Mesothelin is a membrane bound glycoprotein expressed by mesothelial cells and over expressed in malignancy, especially mesothelioma [41]. Soluble mesothelin related proteins (SMRP) are serum proteins thought to be released by alternative splicing of the mesothelin protein and thereby preventing adherence to cell membranes. Serum concentrations are increased in malignant mesothelioma, and therefore this protein is a potential serum marker for the disease [42]. However, although most patients with mesothelioma have raised serum levels of SMRP, giving a sensitivity of between 80–83% and specificity between 80–100% [43-45], it is mostly associated with the epithelioid sub-type, leading to difficulties in identifying the other sub-types of mesothelioma, especially sarcomatoid, using this marker alone [46]. Nevertheless a commercially available FDA-approved assay, MESOMARK (Fujirebio Diagnostics Inc, Malvern, PA, USA), has been recently approved for use in the monitoring of disease in epithelioid and biphasic mesothelioma, and data seems to support its usefulness [47].

Megakaryocyte Potentiating Factor (MPF) is secreted by cells of several mesothelioma cell lines. In recent studies, MPF was elevated in 91% of 56 malignant mesothelioma patients compared with controls, and levels returned to normal after surgery in patients with peritoneal mesothelioma [46,48]. This might make MPF useful in the monitoring of treatment response in mesothelioma.

## Management

Because there can be difficulty reaching a diagnosis of mesothelioma despite radiology, cytology and biopsy, the general management of patients with mesothelioma should be under a multidisciplinary team including respiratory physicians, oncologists, radiologists, palliative care physicians and lung cancer specialist nurses. In addition, once the diagnosis has been reached, there should be close liaison with the patient's primary care physician, and both the primary care physician and family should be warned that, at least in the UK, a post mortem examination will usually be required after the death of a patient with mesothelioma.

The general treatment strategy of mesothelioma should cover the following areas:

• Management of pleural effusions

• Radiotherapy to intervention sites

• Suitability for radical surgery

• Suitability for clinical trial entry and chemotherapy

• Compensation issues

• Palliation of symptoms and end-of-life care

### Management of pleural effusions

Management of malignant pleural effusions begins with therapeutic thoracocentesis, which assesses the response of dyspnoea to fluid removal. If symptoms do not respond to thoracocentesis, alternative causes of dyspnoea should be sought such as pulmonary thromboembolic disease, or lymphangitis carcinomatosis. Early, successful management on pleural effusions with pleurodesis is essential for the palliation of symptoms and the prevention of a trapped lung. However with repeated thoracocentesis, the pleural fluid may undergo loculation making it difficult to drain subsequently and also the risk of pleural infection increases. If a conclusive diagnosis has been made, chemical pleurodesis can be performed via a small bore intercostal chest drain (9–14F), which has equivalent success rates to larger bore chest drains with the added benefit of patient comfort [49,50]. The ideal sclerosing agent is sterile talc, with success rates between 70 and 96% [50-52], although care must be taken to ensure the talc particles are of the optimal calibration to prevent the rare risk of adult respiratory distress syndrome (ARDS) [53].

If a firm diagnosis is yet to be made, and the patient is fit enough for surgery, then thoracoscopy is an extremely useful technique in the management of suspected malignant pleural effusions. This procedure allows visualisation of the pleural surface, enables histological sampling for diagnosis and allows complete drainage of the effusion followed by pleurodesis via talc poudrage.

In patients with a trapped lung, or in whom pleurodesis has failed, a pleuro-peritoneal shunt can be considered. Whilst symptoms can improve in over 90% of patients, complications (including shunt occlusion and infection) occur in 15% and therefore their use is diminishing [54]. More recently, an ambulatory pleural drainage system (Pleur_x _Pleural Catheter, Denver Biomedical Inc, USA) has been developed. This system enables the patient to control the pleural effusion at home by means of a long-term drainage catheter and vacuum bottles. This might help patients with trapped lungs as it both avoids the need for surgical intervention, and palliates dyspnoea [55].

### Radiotherapy

#### Palliative radiotherapy

Radiotherapy can be used to control local tumour growth and occasionally causes regression of disease, but there is no evidence that radiotherapy alone affects survival [56]. It has, however, been shown to be helpful in the palliation of pain [57], and approximately half the patients treated with palliative radiotherapy derive some benefit [58]. It also appears that short courses of radiotherapy (for example 20 Gy in 5 fractions) are as effective as longer courses such as 30 Gy in 10+ fractions [59], although there is a total dose response effect [60]. Unfortunately, radiotherapy is rarely helpful in either the palliation of dyspnoea or the management of symptoms of mediastinal infiltration such as superior vena caval obstruction (SVCO), and alternative methods of treatment such as SVC stenting should be sought [11,61].

#### Radical radiotherapy as a single modality

Whilst hemithorax irradiation may provide symptomatic benefit, no studies have shown that it prolongs survival [61]. Mesothelioma can involve large areas of the pleural cavity and use of radical radiotherapy over large fields places a range of organs such as lungs, liver, spinal cord or heart at a significant risk of dose related damage. As a result, a recent Cochrane review found no randomised clinical trial evidence to support the use of radical radiotherapy alone (or in combination with other treatment modalities) in mesothelioma [62], and this treatment option has been shown to confer a significant mortality, with rates as high as 17% in one series [59].

#### Radiotherapy as part of multimodality treatment

Failure to increase survival using single treatment modalities has led to a multimodality approach to treatment in mesothelioma. Combining debulking surgery with radiotherapy is the cornerstone of this approach and can both reduce systemic recurrence and influence the natural history of the disease [63,64]. Typically, hemithorax radiotherapy is combined with extra pleural pneumonectomy (EPP – see later) and correctly staged patients with epithelioid mesothelioma can have median survival rates of 33 months [65]. Due to the large, irregular nature of malignant mesothelioma along with the close proximity of other organs, a more directed modality of irradiation which could help improve local tumour control whilst limiting exposure to surrounding organs has been developed, known as intensity modulated radiotherapy (IMRT) [66]. Despite the possibilities for improved tumour control, however, IMRT still carries a potential risk of fatal pulmonary toxicity in mesothelioma [67,68].

#### Prophylactic radiotherapy

Mesothelioma may seed malignant cells in procedure scars. Whilst pain from these metastases is rare, they can become uncomfortable, and evidence has suggested that radiotherapy to intervention sites can prevent this complication [69,70]. Whilst this practice is recommended in current guidelines [11], evidence is emerging that it should only be offered to those patients who are symptomatic from these subcutaneous tumours as the radiotherapy itself may have side effects [71,72].

### Surgery

The role of surgery in the treatment of malignant mesothelioma is still uncertain. The three most common surgical procedures are surgical pleurodesis via video assisted thoracoscopic surgery (VATS), debulking surgery (also known as cytoreductive surgery or pleurectomy/decortication (P/D)) and extra pleural pneumonectomy (EPP). This comprises en-bloc resection of the parietal pleura, lung, pericardium and diaphragm and mediastinal nodes [56]. P/D allows the removal of the visceral, parietal and pericardial pleura as well as debulking the tumour and is therefore less demanding, with mortality rates < 5% [73]. P/D has limitations, however, as it does not remove the tumour completely, and the preservation of the ipsilateral lung makes postoperative radiotherapy difficult due to the risk of pulmonary side effects [74]. The impact of P/D on overall survival is contentious, with some evidence suggesting that P/D surgery via a VATS approach may provide a survival benefit in patients with advanced disease not suitable for EPP [75], whilst other studies do not clearly distinguish the benefit of P/D over EPP [76].

EPP is a much more demanding procedure with morbidity rates as high as 60% [77] and mortality rates of 4–9% [11,78,79]. However, the pneumonectomy in EPP allows the use of high dose hemithoracic radiotherapy, and this combined treatment has been shown to reduce local recurrence and prolong survival in early disease [65]. As a result, as part of the trimodality approach to treatment with surgery chemotherapy and radiotherapy, EPP has arisen as the surgical treatment of choice, albeit without clear randomised controlled trial evidence [78]. In order to clarify this issue, a large randomized trial (Mesothelioma And Radical Surgery (MARS)) is underway [80]. In this trial, EPP is sandwiched between induction chemotherapy and radical radiotherapy. The control arm offers full active tri-modality therapy, although the surgery is limited to debulking surgery. In addition patients receive the same induction chemotherapy and are given radiotherapy to any drain or port sites.

### Chemotherapy

The use of chemotherapy in malignant mesothelioma aims to lengthen survival, improve quality of life and provide symptomatic relief. Currently, there is no single drug or combination therapy that could be considered as standard treatment for mesothelioma. A variety of single agent and combination regimens have been tried in clinical trials with response rates of between 0% and 45% [81].

A review of studies by Berghmans *et al *[82] revealed that cisplatin was the most effective single agent for mesothelioma, with carboplatin having similar activity, and in combination with doxorubicin provided a response rate superior to other regimens studied, although there was no clear benefit in terms of survival. Single agent vinorelbine and combination treatment MVP (mitomycin C, vinblastine and cisplatin) have also been shown to provide good symptom relief with acceptable toxicity [83-85].

Recently, there has been much interest in the use of the antifolate pemetrexed (Alimta; Eli Lilly). Pemetrexed exerts its effect by interrupting folate dependent metabolic synthesis of purines and pyrimidines (the building blocks of DNA and RNA), and a Cochrane review of the effectiveness of combination treatment with cisplatin/pemetrexed [81] suggests a survival benefit, albeit with increased toxicity which is reduced by the co-administration of vitamin B12 and folate supplements. Much of the evidence supporting the use of pemetrexed comes from a single study [86] in which 331 patients, fully supplemented with vitamin B12, demonstrated a median survival of 13.3 months with combination pemetrexed/cisplatin compared to 10.0 months with cisplatin alone (*p *= 0.05). This was associated with a significant improvement in quality of life and symptom relief compared to cisplatin alone [87]. Combination with carboplatin instead of cisplatin, however, may be an alternative regimen which provides similar efficacies and reduced side effects [88]. An alternative antifolate agent, raltitrexed, has also been shown to confer a survival benefit in combination with cisplatin, with median overall survival increasing from 8.8 months (CI 7.8–10.8) with cisplatin alone to 11.4 months (CI 10.1 to 15) with combination cisplatin-raltitrexed (*p *= 0.05) [89]. Although this study only demonstrated borderline significance, likely as a result of sample size, the results of these trials mean that combination cisplatin and an antifolate should be the reference regimen in mesothelioma. Recent advances now favour the use of neoadjuvant chemotherapy followed by EPP and radiation for malignant mesothelioma; Weder *et al *recently published their experience of a prospective trial of neoadjuvant chemotherapy consisting of cisplatin and gemcitabine followed by EPP, demonstrating a median survival of 23 months [90]. Similarly, Flores *et al *prospectively studied patients given induction gemcitabine and cisplatin followed by EPP. Median survival of all patients in the study was 19 months, but those patients who completed induction chemotherapy and subsequently underwent EPP had a median survival of 33.5 months [91].

As a result, all patients who are fit enough (ECOG performance status 0–2) should have the opportunity to discuss the merits of chemotherapy in mesothelioma with an oncologist [11], in the knowledge that there are no published data which compares survival or symptom control in patients treated with chemotherapy or best supportive care only. The first such trial (MSO-1) [92] is complete, and the preliminary results were published in abstract form in 2007 [93]. These results suggest that the addition of chemotherapy to active symptom control with best supportive care did not infer a significant survival benefit in mesothelioma, however this trial was started before the emergence of pemetrexed.

### Supportive and palliative care

Given that malignant mesothelioma has a poor overall prognosis, referral of patients to specialist palliative care services will be appropriate at some time in the disease course. Most patients need palliation of symptoms early on in the course of the disease and recognition of this by the patient, family and primary care physician is essential in the management of the patient with mesothelioma. The use of a central lung cancer nurse specialist provides a means by which the patient can gain access to the delivery of care needed for this disease, through the following core elements [11]:

• Communication

• Information

• Coordinated care between respiratory physicians, oncologists, radiologist and palliative care specialists

• Nursing care

• Accessibility

• Support

## Prognosis and survival

Life expectancy in malignant mesothelioma is poor, with median survival varying between 8 and 14 months [11]. The Cancer and Leukaemia Group B, and the European Organization for Research and Treatment of Cancer have analysed large numbers of patients enrolled in treatment trials for mesothelioma and have identified the following poor prognostic factors:

• Non-epithelioid histology

• Poor performance status

• Chest pain

• Age older than 75

• Male gender

• White blood cell count 8.3 × 10^9^/L or greater

• Platelets greater than 400,000 **μ**L

• LDH greater than 500 IU/L

The majority of patients who survive for more than 2 years have epithelioid histology and death from mesothelioma tends to be due to respiratory failure.

## Compensation issues

Eligibility for compensation for mesothelioma may vary from country to country. In the UK for example, a diagnosis of mesothelioma allows compensation via the Industrial Injuries Disablement Benefit, War Pensions Scheme or through a Common Law claim from the firm/firms where the asbestos exposure occurred. Patients who cannot identify occupational exposure to asbestos are not entitled for compensation, however pathological confirmation of the diagnosis is not mandatory; establishing the diagnosis and causation on the balance of probability is sufficient, although an unequivocal diagnosis obviously removes any cause for debate. Earlier this year, a new legislation was made in the UK Child Maintenance and Other Payments Act whereby dependants of persons with mesothelioma became eligible to claim for lump-sum compensation after death .

## Abbreviations

ESR: erythrocyte sedimentation rate; CT: computed tomogram; MRI: magnetic resonance imaging; PET: positron emission tomography; SUV: standardized uptake value; FDG: fluorodeoxyglucose; IMIG: International Mesothelioma Interest Group; WHO: World Health Organization; RECIST: Response Evaluation Criteria in Solid Tumours; EMA: epithelial membrane antigen; SMRP: Soluble mesothelin related proteins; ARDS: adult respiratory distress syndrome; MPF: Megakaryocyte Potentiating Factor; SVCO: superior vena caval obstruction; IMRT: intensity modulated radiotherapy; VATS: video assisted thoracoscopic surgery; P/D: pleurectomy/decortication; EPP: extra pleural pneumonectomy; MARS: Mesothelioma And Radical Surgery.

## Competing interests

The authors declare that they have no competing interests.

## Authors' contributions

All authors contributed to this review article.

## References

[B1] Jett J, Aubry M, Albert, Spiro, Jett Malignant Pleural Mesothelioma. Clinical Respiratory Medicine Mosby.

[B2] Carbone M, Bedrossian CW (2006). The pathogenesis of mesothelioma. Semin Diagn Pathol.

[B3] Wagner JC, Sleggs CA, Marchand P (1960). Diffuse pleural mesothelioma and asbestos exposure in the North Western Cape Province. Br J Ind Med.

[B4] Bianchi C, Giarelli L, Grandi G, Brollo A, Ramani L, Zuch C (1997). Latency periods in asbestos-related mesothelioma of the pleura. Eur J Cancer Prev.

[B5] McElvenny DM, Darnton AJ, Price MJ, Hodgson JT (2005). Mesothelioma mortality in Great Britain from 1968 to 2001. Occup Med (Lond).

[B6] Peto J, Hodgson JT, Matthews FE, Jones JR (1995). Continuing increase in mesothelioma mortality in Britain. Lancet.

[B7] Hodgson JT, McElvenny DM, Darnton AJ, Price MJ, Peto J (2005). The expected burden of mesothelioma mortality in Great Britain from 2002 to 2050. Br J Cancer.

[B8] Marchevsky AM, Wick MR (2003). Current controversies regarding the role of asbestos exposure in the causation of malignant mesothelioma: the need for an evidence-based approach to develop medicolegal guidelines. Ann Diagn Pathol.

[B9] Howel D, Gibbs A, Arblaster L, Swinburne L, Schweiger M, Renvoize E, Hatton P, Pooley F (1999). Mineral fibre analysis and routes of exposure to asbestos in the development of mesothelioma in an English region. Occup Environ Med.

[B10] Yamate J, Tomita A, Kuwamura M, Mitsunaga F, Nakamura S (2007). Spontaneous peritoneal malignant mesothelioma in a geriatric japanese macaque (Macaca fuscata). Exp Anim.

[B11] Wiggins J (2007). BTS statement on malignant mesothelioma in the UK, 2007. Thorax.

[B12] Emri S, Demir A, Dogan M, Akay H, Bozkurt B, Carbone M, Baris I (2002). Lung diseases due to environmental exposures to erionite and asbestos in Turkey. Toxicol Lett.

[B13] Sakellariou K, Malamou-Mitsi V, Haritou A, Koumpaniou C, Stachouli C, Dimoliatis ID, Constantopoulos SH (1996). Malignant pleural mesothelioma from nonoccupational asbestos exposure in Metsovo (north-west Greece): slow end of an epidemic?. Eur Respir J.

[B14] Gibbs GW, Berry G (2008). Mesothelioma and asbestos. Regul Toxicol Pharmacol.

[B15] Testa JR, Carbone M, Hirvonen A, Khalili K, Krynska B, Linnainmaa K, Pooley FD, Rizzo P, Rusch V, Xiao GH (1998). A multi-institutional study confirms the presence and expression of simian virus 40 in human malignant mesotheliomas. Cancer Res.

[B16] Price MJ, Darnton AJ, McElvenny DM, Hodgson JT (2007). Simian virus 40 and mesothelioma in Great Britain. Occup Med (Lond).

[B17] Stenton SC (1997). Asbestos, Simian virus 40 and malignant mesothelioma. Thorax.

[B18] Lopez-Rios F, Illei PB, Rusch V, Ladanyi M (2004). Evidence against a role for SV40 infection in human mesotheliomas and high risk of false-positive PCR results owing to presence of SV40 sequences in common laboratory plasmids. Lancet.

[B19] Manfredi JJ, Dong J, Liu WJ, Resnick-Silverman L, Qiao R, Chahinian P, Saric M, Gibbs AR, Phillips JI, Murray J, Axten CW, Nolan RP, Aaronson SA (2005). Evidence against a role for SV40 in human mesothelioma. Cancer Res.

[B20] Leithner K, Leithner A, Clar H, Weinhaeusel A, Radl R, Krippl P, Rehak P, Windhager R, Haas OA, Olschewski H (2006). Mesothelioma mortality in Europe: impact of asbestos consumption and simian virus 40. Orphanet J Rare Dis.

[B21] Stewart DJ, Edwards JG, Smythe WR, Waller DA, O'Byrne KJ (2004). Malignant pleural mesothelioma – an update. Int J Occup Environ Health.

[B22] Yates DH, Corrin B, Stidolph PN, Browne K (1997). Malignant mesothelioma in south east England: clinicopathological experience of 272 cases. Thorax.

[B23] McGavin C, Hughes P (1998). Finger clubbing in malignant mesothelioma and benign asbestos pleural disease. Respir Med.

[B24] Elmes PC, Simpson JC (1976). The clinical aspects of mesothelioma. Q J Med.

[B25] Selleslag DL, Geraghty RJ, Ganesan TS, Slevin ML, Wrigley PF, Brown R (1989). Autoimmune haemolytic anaemia associated with malignant peritoneal mesothelioma. Acta Clin Belg.

[B26] Armato SG, Entwisle J, Truong MT, Nowak AK, Ceresoli GL, Zhao B, Misri R, Kindler HL (2008). Current state and future directions of pleural mesothelioma imaging. Lung Cancer.

[B27] Leung AN, Muller NL, Miller RR (1990). CT in differential diagnosis of diffuse pleural disease. AJR Am J Roentgenol.

[B28] Okten F, Koksal D, Onal M, Ozcan A, Simsek C, Erturk H (2006). Computed tomography findings in 66 patients with malignant pleural mesothelioma due to environmental exposure to asbestos. Clin Imaging.

[B29] Wang ZJ, Reddy GP, Gotway MB, Higgins CB, Jablons DM, Ramaswamy M, Hawkins RA, Webb WR (2004). Malignant pleural mesothelioma: evaluation with CT, MR imaging, and PET. Radiographics.

[B30] Benard F, Sterman D, Smith RJ, Kaiser LR, Albelda SM, Alavi A (1998). Metabolic imaging of malignant pleural mesothelioma with fluorodeoxyglucose positron emission tomography. Chest.

[B31] Duysinx B, Nguyen D, Louis R, Cataldo D, Belhocine T, Bartsch P, Bury T (2004). Evaluation of pleural disease with 18-fluorodeoxyglucose positron emission tomography imaging. Chest.

[B32] Zervos MD, Bizekis C, Pass HI (2008). Malignant mesothelioma 2008. Curr Opin Pulm Med.

[B33] Rusch VW (1995). A proposed new international TNM staging system for malignant pleural mesothelioma. From the International Mesothelioma Interest Group. Chest.

[B34] Nowak AK (2005). CT, RECIST, and malignant pleural mesothelioma. Lung Cancer.

[B35] Therasse P, Arbuck SG, Eisenhauer EA, Wanders J, Kaplan RS, Rubinstein L, Verweij J, van Glabbeke M, van Oosterom AT, Christian MC, Gwyther SG (2000). New guidelines to evaluate the response to treatment in solid tumors. European Organization for Research and Treatment of Cancer, National Cancer Institute of the United States, National Cancer Institute of Canada. J Natl Cancer Inst.

[B36] Byrne MJ, Nowak AK (2004). Modified RECIST criteria for assessment of response in malignant pleural mesothelioma. Ann Oncol.

[B37] Marchevsky AM (2008). Application of immunohistochemistry to the diagnosis of malignant mesothelioma. Arch Pathol Lab Med.

[B38] Metintas M, Ozdemir N, Isiksoy S, Kaya T, Ekici M, Erginel S, Harmanci E, Erdinc P, Ulgey N, Alatas F (1995). CT-guided pleural needle biopsy in the diagnosis of malignant mesothelioma. J Comput Assist Tomogr.

[B39] Grigoriu BD, Scherpereel A, Devos P, Chahine B, Letourneux M, Lebailly P, Gregoire M, Porte H, Copin MC, Lassalle P (2007). Utility of osteopontin and serum mesothelin in malignant pleural mesothelioma diagnosis and prognosis assessment. Clin Cancer Res.

[B40] Pass HI, Lott D, Lonardo F, Harbut M, Liu Z, Tang N, Carbone M, Webb C, Wali A (2005). Asbestos exposure, pleural mesothelioma, and serum osteopontin levels. N Engl J Med.

[B41] Chang K, Pastan I (1996). Molecular cloning of mesothelin, a differentiation antigen present on mesothelium, mesotheliomas, and ovarian cancers. Proc Natl Acad Sci USA.

[B42] Robinson BW, Creaney J, Lake R, Nowak A, Musk AW, de Klerk N, Winzell P, Hellstrom KE, Hellstrom I (2003). Mesothelin-family proteins and diagnosis of mesothelioma. Lancet.

[B43] Scherpereel A, Grigoriu B, Conti M, Gey T, Gregoire M, Copin MC, Devos P, Chahine B, Porte H, Lassalle P (2006). Soluble mesothelin-related peptides in the diagnosis of malignant pleural mesothelioma. Am J Respir Crit Care Med.

[B44] Robinson BW, Creaney J, Lake R, Nowak A, Musk AW, de Klerk N, Winzell P, Hellstrom KE, Hellstrom I (2005). Soluble mesothelin-related protein – a blood test for mesothelioma. Lung Cancer.

[B45] Pass HI, Wali A, Tang N, Ivanova A, Ivanov S, Harbut M, Carbone M, Allard J (2008). Soluble mesothelin-related peptide level elevation in mesothelioma serum and pleural effusions. Ann Thorac Surg.

[B46] Scherpereel A, Lee YC (2007). Biomarkers for mesothelioma. Curr Opin Pulm Med.

[B47] Beyer HL, Geschwindt RD, Glover CL, Tran L, Hellstrom I, Hellstrom KE, Miller MC, Verch T, Allard WJ, Pass HI, Sardesai NY (2007). MESOMARK: a potential test for malignant pleural mesothelioma. Clin Chem.

[B48] Onda M, Nagata S, Ho M, Bera TK, Hassan R, Alexander RH, Pastan I (2006). Megakaryocyte potentiation factor cleaved from mesothelin precursor is a useful tumor marker in the serum of patients with mesothelioma. Clin Cancer Res.

[B49] Antunes G, Neville E, Duffy J, Ali N (2003). BTS guidelines for the management of malignant pleural effusions. Thorax.

[B50] Heffner JE, Klein JS (2008). Recent advances in the diagnosis and management of malignant pleural effusions. Mayo Clin Proc.

[B51] Shaw P, Agarwal R (2004). Pleurodesis for malignant pleural effusions. Cochrane Database Syst Rev.

[B52] Marom EM, Patz EF, Erasmus JJ, McAdams HP, Goodman PC, Herndon JE (1999). Malignant pleural effusions: treatment with small-bore-catheter thoracostomy and talc pleurodesis. Radiology.

[B53] Maskell NA, Lee YC, Gleeson FV, Hedley EL, Pengelly G, Davies RJ (2004). Randomized trials describing lung inflammation after pleurodesis with talc of varying particle size. Am J Respir Crit Care Med.

[B54] Genc O, Petrou M, Ladas G, Goldstraw P (2000). The long-term morbidity of pleuroperitoneal shunts in the management of recurrent malignant effusions. Eur J Cardiothorac Surg.

[B55] Warren WH, Kalimi R, Khodadadian LM, Kim AW (2008). Management of malignant pleural effusions using the Pleur(x) catheter. Ann Thorac Surg.

[B56] Ceresoli GL, Gridelli C, Santoro A (2007). Multidisciplinary treatment of malignant pleural mesothelioma. Oncologist.

[B57] Davis SR, Tan L, Ball DL (1994). Radiotherapy in the treatment of malignant mesothelioma of the pleura, with special reference to its use in palliation. Australas Radiol.

[B58] Bissett D, Macbeth FR, Cram I (1991). The role of palliative radiotherapy in malignant mesothelioma. Clin Oncol (R Coll Radiol).

[B59] Ball DL, Cruickshank DG (1990). The treatment of malignant mesothelioma of the pleura: review of a 5-year experience, with special reference to radiotherapy. Am J Clin Oncol.

[B60] Gordon W, Antman KH, Greenberger JS, Weichselbaum RR, Chaffey JT (1982). Radiation therapy in the management of patients with mesothelioma. Int J Radiat Oncol Biol Phys.

[B61] Waite K, Gilligan D (2007). The role of radiotherapy in the treatment of malignant pleural mesothelioma. Clin Oncol (R Coll Radiol).

[B62] Chapman E, Berenstein EG, Dieguez M, Ortiz Z (2006). Radiotherapy for malignant pleural mesothelioma. Cochrane Database Syst Rev.

[B63] Allen AM, Den R, Wong JS, Zurakowski D, Soto R, Janne PA, Zellos L, Bueno R, Sugarbaker DJ, Baldini EH (2007). Influence of radiotherapy technique and dose on patterns of failure for mesothelioma patients after extrapleural pneumonectomy. Int J Radiat Oncol Biol Phys.

[B64] Neragi-Miandoab S (2006). Multimodality approach in management of malignant pleural mesothelioma. Eur J Cardiothorac Surg.

[B65] Rusch VW, Rosenzweig K, Venkatraman E, Leon L, Raben A, Harrison L, Bains MS, Downey RJ, Ginsberg RJ (2001). A phase II trial of surgical resection and adjuvant high-dose hemithoracic radiation for malignant pleural mesothelioma. J Thorac Cardiovasc Surg.

[B66] Forster KM, Smythe WR, Starkschall G, Liao Z, Takanaka T, Kelly JF, Vaporciyan A, Ahamad A, Dong L, Salehpour M, Komaki R, Stevens CW (2003). Intensity-modulated radiotherapy following extrapleural pneumonectomy for the treatment of malignant mesothelioma: clinical implementation. Int J Radiat Oncol Biol Phys.

[B67] Rice DC, Smythe WR, Liao Z, Guerrero T, Chang JY, McAleer MF, Jeter MD, Correa A, Vaporciyan AA, Liu HH, Komaki R, Forster KM, Stevens CW (2007). Dose-dependent pulmonary toxicity after postoperative intensity-modulated radiotherapy for malignant pleural mesothelioma. Int J Radiat Oncol Biol Phys.

[B68] Allen AM, Czerminska M, Janne PA, Sugarbaker DJ, Bueno R, Harris JR, Court L, Baldini EH (2006). Fatal pneumonitis associated with intensity-modulated radiation therapy for mesothelioma. Int J Radiat Oncol Biol Phys.

[B69] Boutin C, Rey F, Viallat JR (1995). Prevention of malignant seeding after invasive diagnostic procedures in patients with pleural mesothelioma. A randomized trial of local radiotherapy. Chest.

[B70] Low EM, Khoury GG, Matthews AW, Neville E (1995). Prevention of tumour seeding following thoracoscopy in mesothelioma by prophylactic radiotherapy. Clin Oncol (R Coll Radiol).

[B71] Muirhead R, O'Rourke N (2007). Drain site radiotherapy in malignant pleural mesothelioma: a wasted resource. Eur Respir J.

[B72] O'Rourke N, Garcia JC, Paul J, Lawless C, McMenemin R, Hill J (2007). A randomised controlled trial of intervention site radiotherapy in malignant pleural mesothelioma. Radiother Oncol.

[B73] Rusch VW (1997). Pleurectomy/decortication in the setting of multimodality treatment for diffuse malignant pleural mesothelioma. Semin Thorac Cardiovasc Surg.

[B74] Maasilta P (1991). Deterioration in lung function following hemithorax irradiation for pleural mesothelioma. Int J Radiat Oncol Biol Phys.

[B75] Halstead JC, Lim E, Venkateswaran RM, Charman SC, Goddard M, Ritchie AJ (2005). Improved survival with VATS pleurectomy-decortication in advanced malignant mesothelioma. Eur J Surg Oncol.

[B76] Flores RM, Pass HI, Seshan VE, Dycoco J, Zakowski M, Carbone M, Bains MS, Rusch VW (2008). Extrapleural pneumonectomy versus pleurectomy/decortication in the surgical management of malignant pleural mesothelioma: results in 663 patients. J Thorac Cardiovasc Surg.

[B77] Sugarbaker DJ, Jaklitsch MT, Bueno R, Richards W, Lukanich J, Mentzer SJ, Colson Y, Linden P, Chang M, Capalbo L, Oldread E, Neragi-Miandoab S, Swanson SJ, Zellos LS (2004). Prevention, early detection, and management of complications after 328 consecutive extrapleural pneumonectomies. J Thorac Cardiovasc Surg.

[B78] Treasure T, Sedrakyan A (2004). Pleural mesothelioma: little evidence, still time to do trials. Lancet.

[B79] Sugarbaker DJ, Garcia JP, Richards WG, Harpole DH, Healy-Baldini E, DeCamp MM, Mentzer SJ, Liptay MJ, Strauss GM, Swanson SJ (1996). Extrapleural pneumonectomy in the multimodality therapy of malignant pleural mesothelioma. Results in 120 consecutive patients. Ann Surg.

[B80] Treasure T, Tan C, Lang-Lazdunski L, Waller D (2006). The MARS trial: mesothelioma and radical surgery. Interact Cardiovasc Thorac Surg.

[B81] Green J, Dundar Y, Dodd S, Dickson R, Walley T (2007). Pemetrexed disodium in combination with cisplatin versus other cytotoxic agents or supportive care for the treatment of malignant pleural mesothelioma. Cochrane Database Syst Rev.

[B82] Berghmans T, Paesmans M, Lalami Y, Louviaux I, Luce S, Mascaux C, Meert AP, Sculier JP (2002). Activity of chemotherapy and immunotherapy on malignant mesothelioma: a systematic review of the literature with meta-analysis. Lung Cancer.

[B83] Middleton GW, Smith IE, O'Brien ME, Norton A, Hickish T, Priest K, Spencer L, Ashley S (1998). Good symptom relief with palliative MVP (mitomycin-C, vinblastine and cisplatin) chemotherapy in malignant mesothelioma. Ann Oncol.

[B84] Andreopoulou E, Ross PJ, O'Brien ME, Ford HE, Priest K, Eisen T, Norton A, Ashley S, Smith IE (2004). The palliative benefits of MVP (mitomycin C, vinblastine and cisplatin) chemotherapy in patients with malignant mesothelioma. Ann Oncol.

[B85] Steele JP, Shamash J, Evans MT, Gower NH, Tischkowitz MD, Rudd RM (2000). Phase II study of vinorelbine in patients with malignant pleural mesothelioma. J Clin Oncol.

[B86] Vogelzang NJ, Rusthoven JJ, Symanowski J, Denham C, Kaukel E, Ruffie P, Gatzemeier U, Boyer M, Emri S, Manegold C, Niyikiza C, Paoletti P (2003). Phase III study of pemetrexed in combination with cisplatin versus cisplatin alone in patients with malignant pleural mesothelioma. J Clin Oncol.

[B87] Gralla R, Hollen P, Liepa Aea (2003). Improving quality of life in patients with malignant pleural mesothelioma: results of the randomised pemetrexed + cisplatin trial using the LCSS-meso instrument (abstract). Proc Am Soc Clin Oncol.

[B88] Castagneto B, Botta M, Aitini E, Spigno F, Degiovanni D, Alabiso O, Serra M, Muzio A, Carbone R, Buosi R, Galbusera V, Piccolini E, Giaretto L, Rebella L, Mencoboni M (2008). Phase II study of pemetrexed in combination with carboplatin in patients with malignant pleural mesothelioma (MPM). Ann Oncol.

[B89] van Meerbeeck JP, Gaafar R, Manegold C, van Klaveren RJ, van Marck EA, Vincent M, Legrand C, Bottomley A, Debruyne C, Giaccone G (2005). Randomized phase III study of cisplatin with or without raltitrexed in patients with malignant pleural mesothelioma: an intergroup study of the European Organisation for Research and Treatment of Cancer Lung Cancer Group and the National Cancer Institute of Canada. J Clin Oncol.

[B90] Weder W, Stahel RA, Bernhard J, Bodis S, Vogt P, Ballabeni P, Lardinois D, Betticher D, Schmid R, Stupp R, Ris HB, Jermann M, Mingrone W, Roth AD, Spiliopoulos A (2007). Multicenter trial of neo-adjuvant chemotherapy followed by extrapleural pneumonectomy in malignant pleural mesothelioma. Ann Oncol.

[B91] Flores RM, Krug LM, Rosenzweig KE, Venkatraman E, Vincent A, Heelan R, Akhurst T, Rusch VW (2006). Induction chemotherapy, extrapleural pneumonectomy, and postoperative high-dose radiotherapy for locally advanced malignant pleural mesothelioma: a phase II trial. J Thorac Oncol.

[B92] Muers MF, Rudd RM, O'Brien ME, Qian W, Hodson A, Parmar MK, Girling DJ (2004). BTS randomised feasibility study of active symptom control with or without chemotherapy in malignant pleural mesothelioma: ISRCTN 54469112. Thorax.

[B93] Muers M, Fisher P, O'Brien ME, Peake M, Rudd RM, Snee M, Steele JP, Nankivell M, Pugh C, Stephens R (2007). A randomised Phase III trial of active symptom control with or without chemotherapy in the treatment of patients with malignant pleural mesothelioma. The Medical Research Council/British Thoracic Society MSO 1 Trial [abstract]. Thorax.

